# Cellulose (Nano)Composites

**DOI:** 10.3390/polym15112512

**Published:** 2023-05-30

**Authors:** Denis Mihaela Panaitescu, Adriana Nicoleta Frone

**Affiliations:** National Institute for Research and Development in Chemistry and Petrochemistry—ICECHIM, 202 Splaiul Independentei, 060021 Bucharest, Romania

The environment has been severely affected by the intensive production and use of plastics derived from fossil fuels, and their uncontrolled end-of-life disposal. Recent decades have seen a return to using natural products, and nanocellulose (NC) occupies a privileged position among these intensively studied products [1,2]. NC is obtained from cellulose, the most abundant natural polymer, by applying different chemical, mechanical, enzymatic, and most often, combined methods. A huge effort has been invested in the application of NC as a modifier or reinforcing agent in polymer nanocomposites [1,2]. However, the beneficial effects of NC on the properties of polymer nanocomposites are hindered by the poor dispersion of hydrophilic NC in hydrophobic polymer matrices. The Special Issue ‘Cellulose (Nano)Composites’ brings together twelve original articles and studies that contribute to advances in our collective fundamental and technological knowledge of cellulose-polymer nanocomposites. Isolation of NC from cheap sources and especially from agricultural and forestry waste is an important step to be implemented for cost reduction and environmental protection. NC has been isolated from walnut shells through a mechano-chemical treatment, followed by 2,6,6-Tetramethylpiperidinyloxy (TEMPO) oxidation and ultrasound, showing properties comparable to or better than those of NC obtained by sulfuric acid hydrolysis [3]. Cellulose nanocrystals (CNC) have been extracted from different byproducts of the agro-food industries, such as corncobs, corn husks, wheat bran and coconut shells, and used as reinforcements for chitosan films [4]. Rice straw nanofibers isolated from unbleached pulps had a good effect on the porosity, hydrophilicity, and antifouling of polysulfone membranes [5].

An appropriate surface treatment of NC is a key element for achieving a good interfacial adhesion and superior thermal and mechanical properties in polymer nanocomposites [6,7]. Cellulose has been surface-modified using plasma treatment in liquid, and its effect on the properties of poly(3-hydroxybutyrate) (PHB) was compared to that of TEMPO-oxidized cellulose [6]. In another study, microfibrillated cellulose was first treated with methacryloxypropyltrimethoxysilane, and then graft-polymerized with methacrylic acid, after which it showed better dispersion in a PHB matrix and induced a greater improvement in PHB properties compared to unmodified microfibrillated cellulose [7]. Direct preparation of PHB/CNC nanocomposites using *Cupriavidus necator* fermented in well-dispersed CNC-supplemented culture media was also proposed [8]. CNC was also used as a crosslinker in a poly(acrylic acid-co-acrylamide) multi-responsive composite hydrogel for the controlled release of dyes and drugs [9]. Other methods of expanding the application of NC include more appropriate and green solvent systems for cellulose, the use of biobased plasticizers and toughening agents in NC nanocomposites, or the use of molecular dynamics simulations for the prediction of the compatibility and mechanical properties of cellulose blends [10,11,12].
